# Estimation of TiO_2_-FeO-Na_2_O slag viscosity through molecular dynamics simulations for an energy efficient ilmenite smelting process

**DOI:** 10.1038/s41598-019-53961-1

**Published:** 2019-11-22

**Authors:** Youngjae Kim, Hyunsik Park

**Affiliations:** Mineral Resource Research Division, Korea Institute of Geoscience and Mineral Resources (KIGAM), Daejeon, 34132 Republic of Korea

**Keywords:** Sustainability, Inorganic chemistry

## Abstract

Along with the increasing demand for the TiO_2_ pigment, the ilmenite smelting process has also become significant because it can utilize both rock- and sand-type ilmenite. However, due to the high liquidus temperature of the TiO_2_ slag system, the smelting process is highly energy consuming. In the present study, the viscosity of molten ilmenite slag was estimated using molecular dynamic simulations at a high temperature to achieve an appropriate and efficient slag design. To verify the validity of the simulation technique, experimental measurements were performed in parallel and their results were compared. The effects of FeO and Na_2_O addition on viscosity of TiO_2_ slag were also investigated. The addition of Na_2_O as a flux enhanced the ilmenite smelting process by not only lowering the liquidus temperature but also slowing the drastic viscosity increase. Statistical information obtained from the molecular dynamic simulations revealed a three-dimensional TiO_6_ octahedral network structure. The relationship between viscosity and structural change with varying FeO and Na_2_O concentrations was explored considering the coordination number of Ti and various bonding types.

## Introduction

Both titanium and titanium dioxide (TiO_2_) are widely used worldwide in the production of white pigments, biomaterials, aircraft materials, and construction materials owing to their advantageous properties such as high refractive index, non-toxic characteristics, bio-affinity, high specific strength, and high corrosion resistance. Over 7 million tons of titanium mineral concentrate is produced globally per annum in the mineral form of rutile TiO_2_ and sand- or rock-type ilmenite (FeTiO_3_)^1^. Of these 7 million tons, approximately 94% is utilized for pigments, and the rest is used for titanium metal (3%) and welding rod applications (2%)^2^. According to the U.S. Geological Survey^3^, global consumption of TiO_2_ for the production of white pigments is estimated to increase by 4% annually for the next 10 years. The global titanium metal market is also expected to increase by approximately 7% annually owing to steady growth in its industrial demand for power generation in China and the aerospace industry in the US and EU^4^.

Naturally occurring rutile mineral, which has high TiO_2_ purity of above 95%, is ideal feedstock for TiO_2_ pigment and titanium metal production. However, its limited supply and high price mean that ilmenite ore is extensively used as an alternative^5^. Because ilmenite ore comprises only 30–65% TiO_2_, concentration and beneficiation processes are necessary prior to its use in the production of TiO_2_ pigments and titanium metal. Several processes have been proposed for improving ilmenite ores by separating Fe from TiO_2_. Three methods are generally adopted depending on the type of ilmenite feedstock: the Becher process, the Benelite process, and the ilmenite smelting process. Compared to Becher and Benelite processes, the ilmenite smelting process has an advantage in that most types of ilmenite ore can be applied as feedstock, and Fe is largely collected as a form of pig iron. As a result, the ilmenite smelting process is employed worldwide; its total annual production capacity of TiO_2_ slag is approximately three times that of synthetic rutile produced by the Becher and Benelite processes^6^.

Although the ilmenite smelting process is environmentally friendly and offers process flexibility, it has two major drawbacks: it requires high energy consumption and “upgraded slag process” for achieving high-grade TiO_2_ feed is optionally needed. The ilmenite smelting process is based on carbothermic reduction. During the process, FeO in ilmenite ore is reduced by anthracite and forms molten iron and TiO_2_-concentrated slag. Due to the high liquidus temperature of TiO_2_-concentrated slag, the operating temperature is approximately 1923–1973 K. However, when the amount of FeO in the slag reduces below 9 wt%, there is a drastic increase in the slag liquidus temperature, which causes a sharp increase in electricity consumption. For this reason, 9 wt% FeO is considered the economically critical composition for TiO_2_ slag^7^. As a result, the maximum concentration of TiO_2_ in ilmenite smelting slag is approximately 80–85 wt%.

To achieve a higher concentration of TiO_2_ in the final slag, the addition of a flux has been considered to decrease the liquidus temperature and further reduce the FeO content in the ilmenite slag. Highly reduced slag systems with below 5 wt% FeO have been demonstrated via the addition of a sodium oxide flux at a relatively low bath temperature of approximately 1873 K^8,9^. It should be noted that these studies were performed on pilot scales using carbon crucibles, and the method was not adopted in practice owing to high refractory erosion from alkali attack. However, in 1995, the introduction of a freeze-lining technique to the ferro-alloy smelting process led to innovations in refractory erosion protection^10^. Owing to this technology, various oxides such as K_2_O and B_2_O_3_ have recently been considered as practical flux options for the ilmenite smelting process^11,12^. The addition of a flux into the ilmenite smelting process could innovatively improve the process conditions and energy efficiency by drastically lowering the operating temperature.

The addition of flux into ilmenite slag affects not only the liquidus temperature but also the thermophysical properties of the slag. Viscosity is one of the most crucial properties because it is closely related to the slag foaming characteristic, the kinetics of the reduction reaction, and the effective separation of the slag and metal^13,14^. However, in spite of its practical importance, only a few experimental studies have investigated the viscosity of ilmenite smelting slag systems, and only with limited slag composition ranges^15–18^. Due to the extremely high melting temperature of high-TiO_2_ slag systems, experimental measurements are challenging. Thus, to properly design an ilmenite slag system considering the effects of flux additions, a prediction method for the viscosity of the TiO_2_–FeO-based system should be established.

In the present study, the viscosities of the FeO–TiO_2_ binary system and the FeO–TiO_2_–Na_2_O ternary system were measured experimentally, and the structures and viscosities were estimated using molecular dynamics (MD) simulations. Thus, the calculated viscosities were compared with the experimental results. After confirming the validity of the potential function used in the MD simulations, the effect of a high concentration of Na_2_O on the viscosity of the TiO_2_–FeO system was estimated. The simulations suggest that a large addition of Na_2_O can achieve an energy-efficient ilmenite smelting process at relatively low temperature. Finally, the effect of the TiO_6_ octahedral network structure on viscosity was investigated by using statistical structure information obtained from the MD simulations.

## Results and Discussion

### Effect of temperature and composition on viscosity

**Figure 1:**
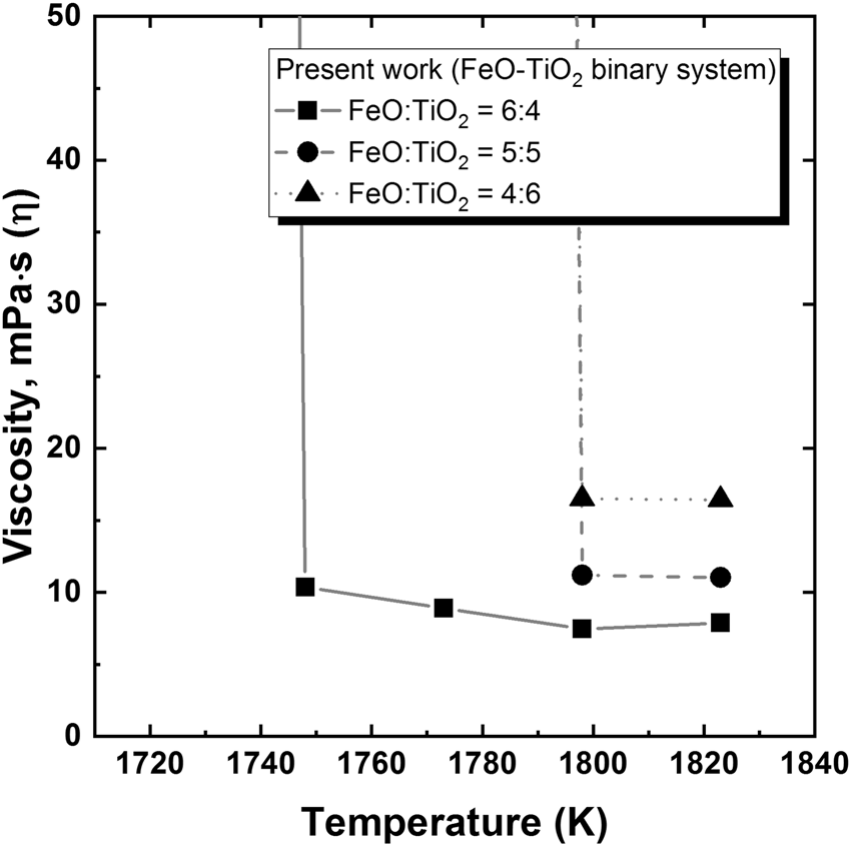
Temperature dependence of viscosity in the molten FeO–TiO_2_ systems. FeO/TiO_2_ = ■ 6:4, ● 5:5, ▲ 4:6.

**Figure 2:**
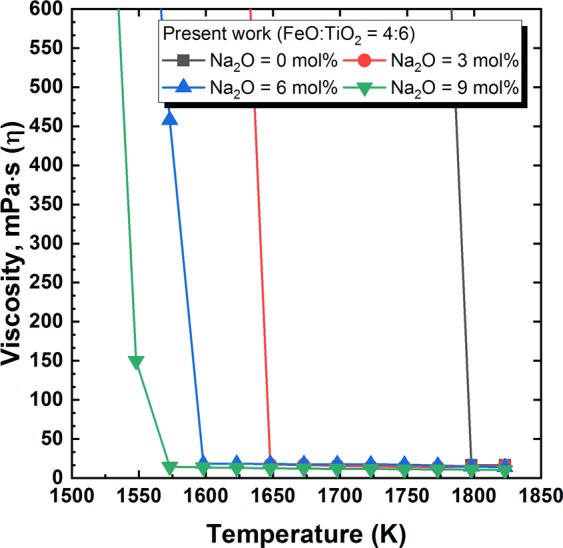
Temperature dependence of viscosity in the molten 2FeO–3TiO_2_–Na_2_O systems. NaO_2_ mol% = ■ 0,  3,  6,  9.

Figure 1 shows the temperature dependence of viscosity on the varying FeO/TiO_2_ ratio. (The measured viscosity values of present study are listed in Supplementary Table S1) Compared to other iron and steel making slag systems with viscosities of approximately 2 dPa∙s at 1823 K, the present FeO–TiO_2_-based slag systems had extremely low viscosities. In addition, compared to the conventional silicate-based steelmaking slag system, the effect of temperature on viscosity was relatively small in the FeO–TiO_2_ system above its liquidus temperature. Because the Arrhenius-type plot of inverse absolute temperature and logarithm of viscosity indicates the activation energy, the present finding implies a minute structural change in the TiO_2_-based network system as temperature varies. However, drastic viscosity increases were observed below the liquidus temperature. Since slag foaming is directly related to the slag viscosity^19^, the abrupt increase in viscosity; that is the lack of slag fluidity, caused sudden and massive slag foaming during the ilmenite smelting process. Because such uncontrollable massive froth formation forces a furnace shutdown^15^, the operation should be carried out and precisely controlled at a temperature sufficiently higher than the liquidus temperature. The effect of Na_2_O addition on the temperature dependence of viscosity in the 2FeO–3TiO_2_ system is shown in Fig. 2. A greater addition of Na_2_O resulted in a lower liquidus temperature. Even a small addition of Na_2_O (3 mol%) drastically decreased the liquidus temperature by approximately 150 K. With more than 6 mol% Na_2_O, a relatively moderate increase in viscosity near the liquidus temperature was observed, which would prevent massive surges of slag foaming during actual operation. According to thermodynamic calculations by using FactSage 7.0 (Thermfact and GTT-Technologies, Motreal, Canada), drastic decreasing of liquidus temperature can be observed as Na_2_O addition in the FeO-TiO_2_ system. In addition, the relative fraction of pseudobrookite phase; that is the primary solidification phase, decreases as increasing of Na_2_O at below its liquidus temperature. According to Wang *et al*.^20^ who found the relationship between thermodynamics and kinetics of Bain transition in pure iron, the decrease of driving force (│*ΔG*│) for phase transformation is correlated with the increase of energy barrier (*Q*). It could be inferred that the decreasing of driving force (│*ΔG*│); that is thermodynamic parameter, results in the slowing kinetics by increasing energy barrier (*Q*). In the present study, the thermodynamic calculations revealed that the addition of Na_2_O restrains the formation of pseudobrookite phase by decreasing of its Gibbs free energy change (│*ΔG*│). As a result, it would be inferred that the energy barrier (*Q*) of phase transformation increases with higher concentration of Na_2_O in the FeO-TiO_2_ slag system. Therefore, the lowering liquidus temperature by decreasing of Gibbs free energy change (│*ΔG*│) of the formation of psedobrookite phase results in the slowing the drastic increasing of viscosity by increasing of energy barrier (*Q*) of phase transformation as higher concentration of Na_2_O.

Therefore, it can be expected that the addition of Na_2_O as a flux into the ilmenite smelting process could enhance the operation conditions and energy efficiency. Considering the lowering liquidus temperature and reducing of the drastic increasing of viscosity, approximately 6 mol% of Na_2_O addition would be suitable for the better operation in the ilmenite smelting process. Since the addition of Na_2_O decreases the liquidus temperature of slag system, a further reduction of FeO can be expected under the same operation conditions. According to previous studies^8,9^, highly reduced TiO_2_ slag with below 5 wt% FeO can be obtained by adding Na_2_O as flux in the ilmenite smelting process. Since the ilmenite smelting slag contains approximately 10 wt% FeO, a further 5 wt% reduction of FeO would result in an approximately 5% increase in pig iron production.

**Figure 3:**
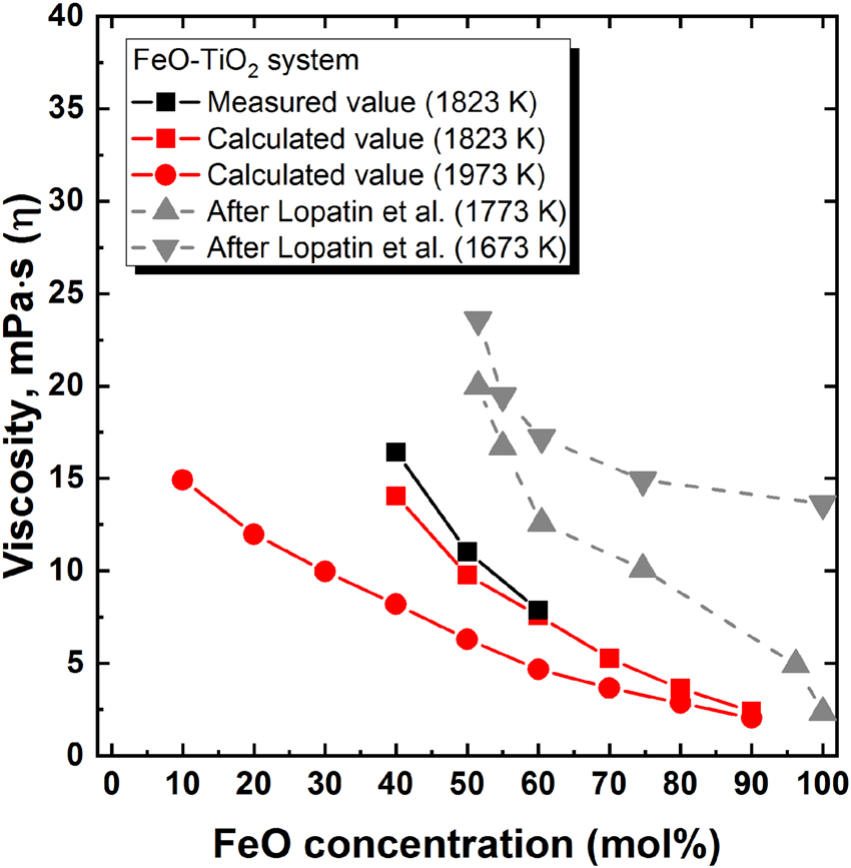
Measured and calculated viscosities of FeO–TiO_2_ binary system with varying FeO concentrations at 1823 and 1973 K. ■ Measured at 1823 K;  Calculated at 1823 K;  Calculated at 1973 K;  Lopatin *et al*.^21^ at 1773 K;  Lopatin *et al*.^21^ at 1673 K.

**Figure 4:**
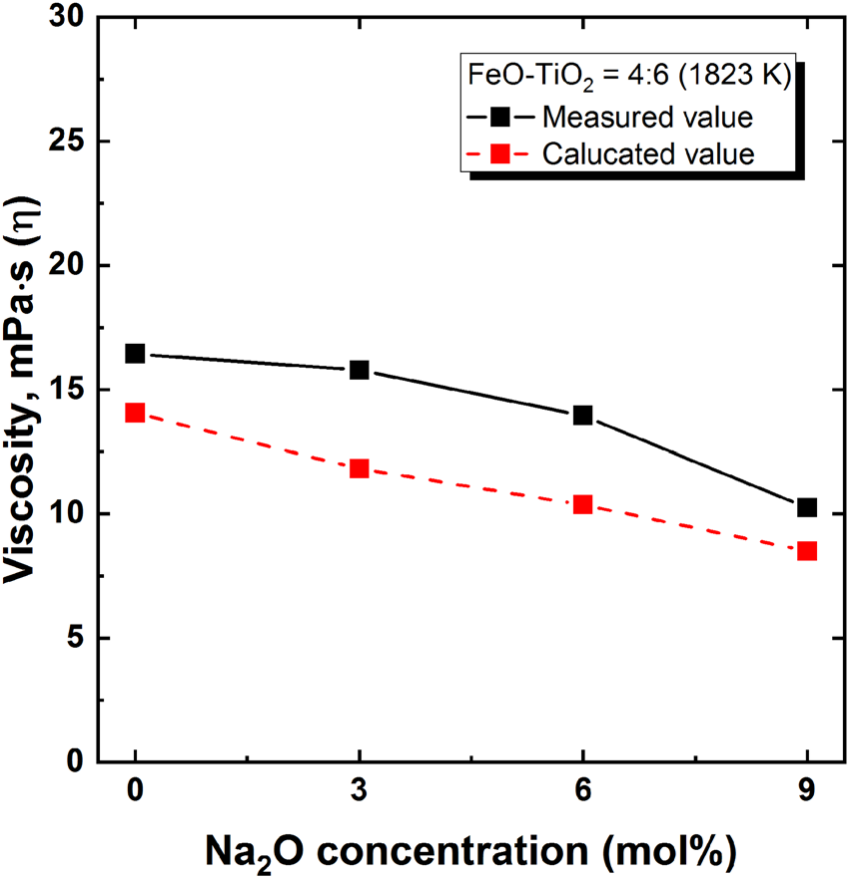
Effect of Na_2_O addition on measured and calculated viscosities of 2FeO–3TiO_2_ system at 1823 K. ■ Measured;  Calculated.

Figure 3 shows the effect of FeO concentration on the viscosity of the FeO–TiO_2_ system. A higher concentration of FeO resulted in a decreased viscosity. A similar dependency of viscosity on FeO concentration was demonstrated in a previous work^21^. In that study, the ferrous (Fe^2+^) to ferric (Fe^3+^) ratio was approximately 10, which is similar to in the present system. Nevertheless, in spite of the similar oxygen potential conditions, the previous work^21^ was carried out at 1773 and 1673 K, which is lower than the liquidus temperature of FeO–TiO_2_^22^. It seems that the decreased liquidus temperature may have resulted from the presence of uncontrolled impurities during their measurements. As shown in Fig. 4, the Na_2_O addition also decreased the viscosity of the 2FeO–3TiO_2_ system. However, compared to the drastic effect on liquidus temperature, the effect on viscosity is relatively minor.

Even in an equilibrium state, atoms and molecules are constantly moving. This collective motion of particles is called Brownian motion, which can be described by the self-diffusion coefficient (*D*). Each atom has its own *D* value. During the MD simulation, *D* can be obtained from the mean square displacement (MSD), that is, the average displacement of a target atom during a certain time *t*, as given by Eq. (1) ^23^.

1 $$MSD=\langle {\Delta }^{2}r(t)\rangle =\frac{1}{N}\mathop{\sum }\limits_{i=1}^{N}\langle \,{[{r}_{i}(t)-{r}_{i}(0)]}^{2}\rangle ,$$

where *r*_*i*_(*t*) is the position of atom *i* at time *t*, and *N* is the number of atoms. The angular brackets indicate the average over atomic positions^23^.

Following Einstein’s relations for diffusivity, the value of *D* for each species can be calculated based on the obtained MSD as expressed in Eq. (2) ^23–27^.

2 $$D=\frac{1}{6}{\mathrm{lim}}_{t\to \infty }\frac{\langle {\Delta }^{2}r(t)\rangle }{t}.$$

Finally, viscosity (*η*) can be calculated from the relationship between *η* and *D* by the Stokes–Einstein equation given in Eq. (3) ^25,27^:

3 $$\eta =\frac{kT}{3\pi \lambda D},$$

where *k* is Boltzmann’s constant, *T* is absolute temperature (*K*), and *λ* is the jumping distance (Å) during diffusion. In the present calculation, the jumping distance was set as the diameter of oxygen (2.8 Å)^27^.

**Figure 5:**
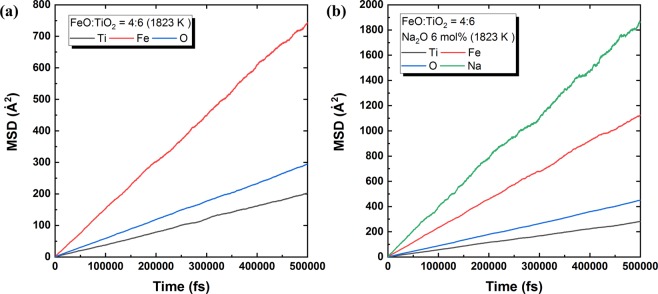
MSDs of Ti (black), Fe (red), O (blue), and Na (green) as a function of time (fs) in the **(a)** 2FeO–3TiO_2_ system with no Na_2_O addition and **(b)** 2FeO–3TiO_2_ system with 6 mol% Na_2_O at 1823 K.

Figure 5 shows the obtained MSDs for the present FeO–TiO_2_ and 2FeO–3TiO_2_–Na_2_O systems at 1823 K. Depending on atomic species, different MSD slopes were obtained as a function of time (fs). Across the studied FeO–TiO_2_-based systems, Ti atom diffusion was commonly the slowest because TiO_2_ forms a TiO_6_ network structure. Thus, the diffusion of Ti atoms plays a determining role in the rheological behaviour of the present system. For this reason, viscosity was calculated based on the MSD of Ti atoms. Further discussion regarding the TiO_2_ structure is given in the following section.

As shown in Figs 3 and 4, the calculated viscosities were in excellent accordance with the measured viscosities, indicating the validity of the employed MD calculations for the FeO–TiO_2_ and 2FeO–3TiO_2_–Na_2_O systems. In addition, the viscosity of the FeO–TiO_2_ system at 1973 K with varying compositions was evaluated based on the present MD simulation, as shown in Fig. 3. Although the results of the MD calculation for viscosity should be verified at high temperature (1973 K) to ensure accurate viscosity modelling, the present results suggest the possibility of an efficient viscosity estimation, even at high temperatures where experimental measurements are challenging. This simple and reliable estimation of viscosity would be expected to aid in the design of slag systems for the ilmenite smelting process. It should be noted that Ti^3+^ also exists in ilmenite smelting slag (Ti^3+^/Ti^4+^ = 0.29) due to its extreme reducing conditions^28^. Such extremely reducing conditions cannot be achieved in our study; thus, Ti^4+^ is in a thermodynamically stable state herein. For this reason, only Ti^4+^ was considered in our MD simulation. However, for the accurate prediction of viscosity in the real ilmenite smelting process, Ti^3+^ should be considered in the MD simulation.

### Effect of titanate structure on viscosity

**Figure 6:**
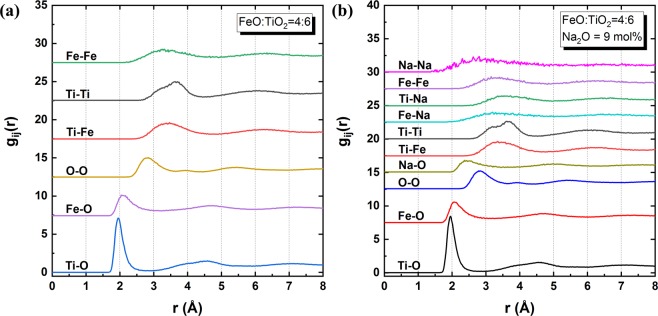
RDFs from MD simulations of the **(a)** 2FeO–3TiO_2_ system with no Na_2_O (Fe–Fe, green; Ti–Ti, grey; Ti–Fe, red; O–O, orange; Fe–O, purple; Ti–O, blue) and **(b)** the 2FeO–3TiO_2_ system with 9 mol% Na_2_O (Na–Na, pink; Fe–Fe, purple; Ti–Na, green; Fe–Na, light blue; Ti–Ti, grey, Ti–Fe, dark red; Na–O, yellow-green; O–O, blue; Fe–O, red; Ti–O, black) at 1823 K.

Figure 6 shows the radial distribution functions (RDFs, *g*_*ij*_(*r*)) of the 2FeO–3TiO_2_ system and 2FeO–3TiO_2_ with 9 mol% Na_2_O system at 1823 K obtained from the MD simulations. The RDF describes the average number density of particles *j* from a reference particle *i* as a function of distance *r*. Relatively sharp peaks were observed at distances less than 2.8 Å between Ti–O, Fe–O, O–O, and Na–O, whereas broadened peaks with gentle slopes were present in other combinations at distances greater than approximately 3.0 Å. Therefore, it can be inferred that meaningful bonding only formed between cations and oxygen or between oxygen and oxygen, which is similar to other silicate-based slag systems.

**Figure 7:**
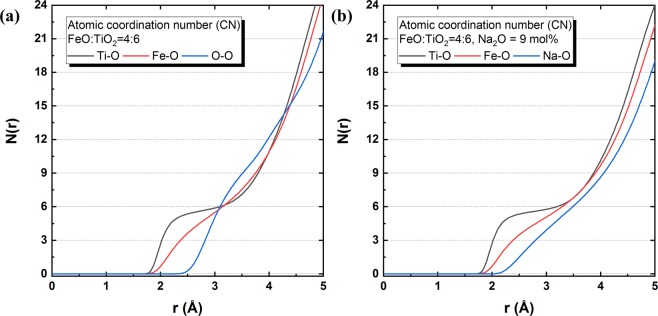
Coordination numbers of Ti, Fe, O and Ti, Fe, Na bonded to oxygen in the **(a)** 2FeO-3TiO_2_ system without Na_2_O and **(b)** the 2FeO-3TiO_2_ system with 9 mol% Na_2_O at 1823 K. Ti–O, black; Fe–O, red; Na–O, blue.

Via integration of the RDF from 0 to a cut-off radius, the coordination numbers (CNs, *N*(*r*)) of each atomic species bonded to oxygen were calculated using the MD simulation results. As shown in Fig. 7, the coordination numbers of Ti, Fe, O, and Na atoms were considered because they have meaningful bonds with O atoms in the present system. A flat plateau was found only for Ti–O, indicating that the coordination number of Ti was approximately 6. Thus, each Ti atom forms an octahedral structure bonded to  six-oxygen atoms. On the other hand, the coordination number curves of other atoms did not show characteristic plateau regions, but instead simply increased at distances greater than 2 Å. Although Fe–O and Na–O formed meaningful bonds, they did not exhibit a regular structural unit. Hence, in the FeO–TiO_2_ and 2FeO–3TiO_2_–Na_2_O systems, the TiO_6_ octahedral structure is the main structural unit, and Fe or Na would compensate for the ionic charge of the TiO_6_ octahedral unit.

Although the formation of a TiO_6_ octahedral structure in the FeO–TiO_2_ slag system has previously been reported^13,29,30^, the formation of a three-dimensional network structure by TiO_6_ octahedra has not yet been clearly verified. Considering that TiO_2_ forms a three-dimensional network structure via linking of TiO_6_ octahedral units in rutile TiO_2_ and ferrous-pseudobrookite (FeTi_2_O_5_) crystals^29^, the formation of a three-dimensional network structure in the molten state should be considered. To evaluate the existence of a three-dimensional network structure consisting of TiO_6_ octahedral units, bond angle distributions (BADs) of O–Ti–O and Ti–O–Ti were investigated. In the present study, BADs were calculated using the interactive structure analysis of amorphous and crystalline systems (ISAACS) program^31^.

**Figure 8:**
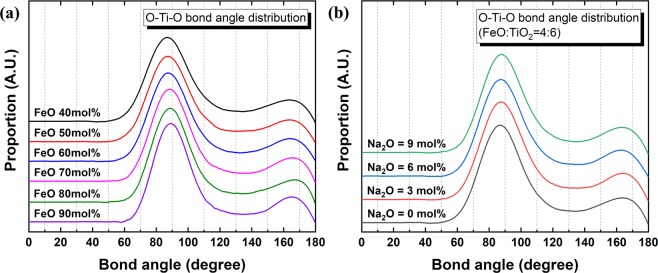
O-Ti-O BAD with varying FeO and Na_2_O concentrations in the **(a)** FeO–TiO_2_ (FeO mol% = 40, black; 50, red; 60, blue; 70, pink; 80, green; 90, purple) and **(b)** 2FeO–3TiO_2_–Na_2_O (Na_2_O mol% = 9, green; 6, blue, 3, red; 0, black) systems at 1823 K.

**Figure 9:**
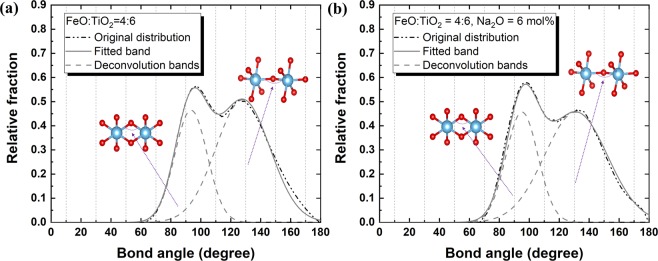
Ti–O–Ti BAD in the **(a)** 2FeO–3TiO_2_ system without Na_2_O and **(b)** 2FeO–3TiO_2_ with 6 mol% Na_2_O at 1823 K. Light blue atoms, Ti; red atoms, oxygen. The deconvolution bands centred at 95° and 130° represent the edge-sharing and corner-sharing TiO_6_ octahedral units, respectively.

**Figure 10:**
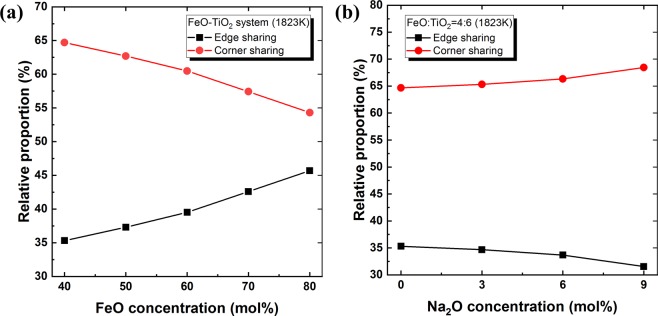
Relative proportion (%) of edge- (■) and corner-sharing () TiO_6_ octahedral units with various FeO and Na_2_O concentrations in the **(a)** FeO–TiO_2_ and **(b)** 2FeO–3TiO_2_–Na_2_O systems at 1823 K.

The O–Ti–O BAD is shown in Fig. 8, displaying distinct peaks at approximately 90° and 170° that indicate the presence of a TiO_6_ octahedral structure in the present system. The much higher peak observed at 90° than at 170° suggests a slightly distorted octahedral structure^24^. The compositional change does not result in a significant bond angle change, implying a stable structure of the TiO_6_ octahedral unit. The O–Ti–O BAD is based on the bond angle between two O atoms on a centre Ti atom in the TiO_6_ octahedral unit. On the other hand, the Ti–O–Ti BAD indicates the bonding between two Ti atoms linked through one or two O atoms. Therefore, the existence of a regular Ti–O–Ti BAD confirms a three-dimensionally linked network structure of TiO_6_ octahedral units in the present system. As shown in Fig. 9, two distinctive broad peaks are centred at 95° and 130° in the Ti–O–Ti BAD, representing the edge-sharing and corner-sharing bond angles, respectively^27,32^. Following the Gaussian deconvolution of the obtained Ti–O–Ti BAD peaks, the relative fractions of edge-sharing and corner-sharing structural units were determined based on the peak area ratios. As shown in Fig. 10, the relative fraction of edge-sharing units gradually decreases as TiO_2_ concentration increases. Because two octahedral units are linked via one sharing O atom, corner sharing has a lower bond strength than edge sharing^27^. However, in spite of the decreasing relative proportion of edge sharing, increasing viscosity was observed at higher TiO_2_ concentrations in the present FeO–TiO_2_ system. In addition, although viscosity decreased at higher Na_2_O concentrations, the relative proportion of edge and corner sharing did not significantly change. Therefore, these results indicate that the type of connection between two octahedral units of TiO_6_ is not directly related to its rheological behaviour.

**Figure 11:**
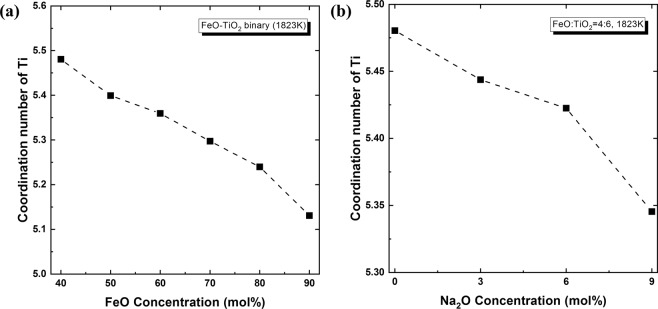
Change in Ti coordination number with varying FeO and Na_2_O concentrations in the **(a)** FeO–TiO_2_ and **(b)** 2FeO–3TiO_2_–Na_2_O systems at 1823 K.

Figure 11 shows the change in coordination number of the Ti atom with varying FeO and Na_2_O concentrations. Further addition of FeO or Na_2_O to the FeO–TiO_2_ system leads to a decrease in coordination number from 5.5 to 5.1 or 5.5 to 5.35, respectively. Considering that the coordination number of Ti–O represents that average bonding of Ti atoms to O atoms, a decrease in coordination number implies the breaking of bonds between them. Since the TiO_6_ octahedral structure forms a network structure in the FeO–TiO_2_ slag system, the decrease of coordination number therefore indicates the depolymerization of TiO_6_ network structure via the formation of non-bridging oxygen (NBO). Thus, due to the network-modifying characteristics of FeO and Na_2_O^33^, their addition depolymerizes the TiO_6_ network structure. After modifying the TiO_6_ octahedral network structure, Fe^2+^ and Na^+^ ions act as ion compensators for NBO^34^. This is supported by a relatively sharp peak observed in the RDF (Fig. 6), implying a regular coupling between Fe^2+^/Na^+^ and O atoms.

**Figure 12:**
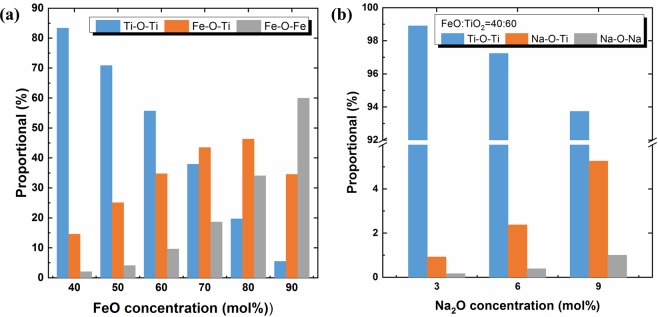
Relative proportions (%) of **(a)** Ti–O–Ti, Fe–O–Ti, and Fe–O–Fe bonds in the FeO-TiO_2_ binary system with various concentrations of FeO and **(b)** Ti–O–Ti, Na–O–Ti, and Na–O–Na bonds in the 2FeO–3TiO_2_–Na_2_O system with various concentrations of Na_2_O at 1823 K.  Ti–O–Ti;  (Fe/Na)–O–Ti;  (Fe/Na)–O–(Fe/Na).

In Fig. 12, the relative proportion of the three bond types (Ti–O–Ti, Fe–O–Ti, and Fe–O–Fe in the FeO–TiO_2_ system, and Ti–O–Ti, Na–O–Ti, and Na–O–Na in the 2FeO–3TiO_2_–Na_2_O system), are described based on the MD simulation results at 1823 K. As shown in Fig. 12(a), a gradual decrease in the occurrence of Ti–O–Ti bonding is observed at higher concentrations of FeO, which indicates depolymerization of the TiO_6_ octahedral structure. In the FeO–TiO_2_ binary system, the occurrence of Fe–O–Ti bonding initially increases but decreases upon reaching 80 mol% FeO. In contrast, the occurrence of Fe–O–Fe bonding drastically increases above 90 mol% FeO. As the TiO_6_ octahedral structure is depolymerized by the FeO addition, the bonding between Fe, O, and Ti increases as a result of the charge compensation of NBO by Fe^2+^. However, above 90 mol% of FeO, the main structure is not a TiO_6_ octahedral unit but rather an FeO-based structure, although as shown in the coordination number curve in Fig. 7, it seems that FeO does not form a regular structural unit in the FeO–TiO_2_ system. Along with depolymerization of TiO_6_ structural units by FeO, FeO also forms Fe–O–Fe bonds based on the charge potential.

As shown in Fig. 12(b), addition of Na_2_O similarly results in decreased Ti–O–Ti bonding, indicating the depolymerization of the TiO_6_ octahedral structure. The increased relative proportion of Na–O–Ti bonds with higher concentrations of Na_2_O arises from the charge compensation of NBO by Na^+^ in the TiO_6_ structure. A small increase in the occurrence of Na–O–Na bonding implies that not all Na_2_O is consumed to depolymerize the TiO_6_ units, but that some Na_2_O naturally forms an ionic bond based on its potential in the molten 2FeO–3TiO_2_–Na_2_O system.

These structural investigations allow for an explanation of the observed viscosity change with varying FeO and Na_2_O concentrations. As the FeO and Na_2_O concentrations increase, the coordination number of Ti atoms bonded to O atoms gradually decreases, indicating the depolymerization of the three-dimensional TiO_6_ network structure. As a result, the rheological behaviour of the molten oxide system improves and viscosity decreases. The decrease in the relative proportion of Ti–O–Ti bonds and increase in proportions of Fe–O–Ti and Fe–O–Fe or Na–O–Ti and Na–O–Na bonds confirmed the breaking of the titanate network and charge compensation of NBO.

## Conclusions

To achieve an energy-saving and efficient ilmenite smelting process, this study considered a slag design with a lowered liquidus temperature via flux addition. MD simulations adopting Buckingham potentials were carried out to estimate ilmenite smelting slag viscosities at high temperatures. To verify the validity of the present simulation, calculations were performed on the relevant crystals (rutile, ilmenite, and Freudenbergite) at room temperature in advance. In addition, the viscosity of the FeO–TiO_2_-based slag system was experimentally measured at 1823 K for comparison with the calculated viscosities. Following the verification of the simulation potential, viscosities were estimated with varying concentrations of TiO_2_ at 1823 and 1973 K. In addition, the effect of Na_2_O as a flux on the ilmenite smelting process was studied. The viscosity measurement results indicated that the addition of Na_2_O enhances the operation conditions by not only lowering the liquidus temperature but also by slowing the drastic increase in viscosity. The addition of Na_2_O is thus expected to reduce the electrical consumption and prevent massive surges of slag foaming during the ilmenite smelting process. Furthermore, the calculated viscosities were in excellent accordance with the measured values, indicating the applicability of MD simulations for slag design in the ilmenite smelting process. By analysing the statistical information obtained from the MD simulation results, the existence of a three-dimensional network structure consisting of TiO_6_ octahedral units was verified. Increased FeO and Na_2_O concentrations were confirmed to depolymerize the titanate network structure by forming NBO, resulting in a decreased Ti coordination number and relative proportion of Ti–O–Ti bonds. In addition, Fe^2+^ and Na^+^ act as charge compensators, forming Fe–O–Ti and Na–O–Ti or Fe–O–Fe and Na–O–Na bonds.

## Methods

### Viscosity measurements

Reagent-grade ilmenite (FeTiO_3_), TiO_2_, FeO, and Na_2_CO_3_ were mixed to obtain target mole ratios. Measurements were carried out with up to 60 mol% TiO_2_ because the liquidus temperature exceeds 1873 K above 65 mol% TiO_2_^22^, and such a high temperature cannot be stably maintained in our present measurement system. The mixtures were ground in an agate mortar for homogeneous mixing and put into a crucible (outer diameter *φ* = 41 mm, inner diameter *φ* = 40 mm, and height = 65 mm) and placed in a vertical furnace at 1573 K under an Ar atmosphere. After heating the sample to 1823 K and holding for 2 h, the viscosity measurement was carried out by introducing spindle at the centre of the molten oxide. In order to prevent chemical erosion of the crucible and spindle from the molten slag system, a Pt–10%Rh alloy was chosen as the material for the crucible and spindle.

The immersed spindle was rotated at 80 rpm, and the measured shear stress was recorded each second by a digital viscometer (DV2TLV; Brookfield Engineering Laboratories, Middleboro, MA) calibrated with standard silicone oil at room temperature. After a 1 h measurement, the temperature was decreased by 25 K at 2.5 K/min. After 20 min of thermal equilibration time, the viscosity measurement was again carried out. This process of decreasing the temperature and measuring the viscosity was continued until the viscosity exceeded 10 Pa∙s. Once the viscosity exceeded 10 Pa∙s, the measurement was ceased, and the sample was reheated to 1823 K. After holding at 1823 K for 1 h, the fully melted sample was quenched on a water-cooled copper plate. The chemical composition of the obtained sample was analysed by using inductively coupled plasma-optical emission spectroscopy (ICP-OES, 5300DV, Perkin Elmer, NY, USA), and the results are listed in Table 1. The ferric (Fe^3+^) and ferrous (Fe^2+^) states of the system were analysed by using the K_2_Cr_2_O_7_ titration method (JIS M 8212:2005).

**Table 1:** Initial and final compositions of the viscosity measurement samples.

(mol%)	Initial composition			Final composition				
	Fe_t_O	TiO_2_	Na_2_O	FeO	Fe_2_O_3_	TiO_2_	Na_2_O	Fe^2+^/(total Fe)
FT46	40.0	60.0		31.9	3.0	65.1		0.91
FT55	50.0	50.0		46.6	2.7	50.7		0.95
FT64	60.0	40.0		54.1	3.0	42.9		0.95
FT46N3	38.8	58.2	3.0	32.7	2.4	62.2	2.7	0.93
FT46N6	37.6	56.4	6.0	24.6	6.4	63.8	5.2	0.79
FT46N9	36.4	54.6	9.0	23.6	6.8	60.1	9.4	0.78

### Molecular dynamics simulations

In the present study, the Buckingham potential was adopted to express the pair interatomic interaction between the cation and oxygen. The Buckingham potential, *E*_*ij*_(*r*), consists of two components as shown in Eq. (4), describing a short-range repulsive interaction and an attractive van der Waals force:

4 $${E}_{ij}(r)={A}_{ij}\exp (-r/{\rho }_{ij})-{C}_{ij}/{r}^{6},$$

where *r* is the interaction distance, *A*, *ρ*, and *C* are potential parameters, and *i* and *j* indicate different atomic species^35^. The potential parameters for Ti, Fe, Na, and O atoms were obtained from Guillot and Sotor’s work^36^ because its applicability for the entire range of industrial oxide systems has been well proven^23^. The detailed atomic charges and Buckingham potential parameters are listed in Table 2. The cation–cation interactions were set to zero, because cation–oxygen and oxygen–oxygen interactions are the dominant factors in the oxide system at short ranges^36,37^.

**Table 2:** Potential parameters adopted in the MD simulations^36^.

Element	Partial charge (e)	Parameters of the interatomic potential			
		Bond	*A*_*ij*_ (ev)	*ρ*_*ij*_ (Å)	*C*_*ij*_ (eV·Å^6^)
O	−0.945	O–O	9022.79	0.265	85.0921
Fe^2+^	0.945	Fe^2+^–O	13032.93	0.19	0
Ti	1.89	Ti–O	50126.64	0.178	46.2978
Na	0.4725	Na–O	120303.8	0.17	0

Generally, MD simulation results of glass systems are compared with neutron structure factors obtained experimentally via neutron scattering to evaluate the validity of the applied potential and calculation procedure. However, due to the highly crystalline characteristics of the FeO–TiO_2_-based system, its glass neutron structure factor cannot be directly obtained. Thus, the experimental and calculated lattice parameters of related crystals were compared to evaluate the accuracy of the Buckingham potential for the FeO–TiO_2_ and FeO–TiO_2_–Na_2_O systems. Three crystal phases were considered: rutile (TiO_2_), ilmenite (FeTiO_3_), and Freudenbergite (Na(Ti_3_Fe)O_8_). As shown in Table 3, the deviations between measured^38–40^ and calculated lattice parameters were less than 3.6%, indicating the reliability of the presented potential and calculation conditions.

**Table 3:** Calculated and measured^38–40^ lattice parameters of FeO, TiO_2_, and Na_2_O containing crystal phases.

Crystal		a (Å)	b (Å)	c (Å)	α (degree)	β (degree)	γ (degree)
Rutile (TiO_2_)	Measured^38^	4.5937	4.5937	2.9587	90.0	90.0	90.0
	Calculated	4.6651	4.6651	3.0047	90.0	90.0	90.0
Ilmenite (FeTiO_3_)	Measured^39^	5.09	5.09	14.09	90.0	90.0	120.0
	Calculated	5.25	5.25	14.52	90.0	90.0	120.0
Freudenbergite (Na(Ti_3_Fe)O_8_)	Measured^40^	12.27	3.82	6.48	90.0	107.16	90.0
	Calculated	12.71	3.96	6.72	90.0	107.16	90.0

A classical MD simulation was carried out in three dimensions with approximately 8,000 atoms. To simplify the simulation, only the ferrous state of iron was considered. Because the ferrous to ferric ratio is more than 10, the effect of the ferric state on the present system would be insignificant. Randomly distributed atoms were generated by using the PACKMOL package^41^ in a fixed cubic box, and the LAMMPS MD code^42^ was applied for the MD simulations. To calculate the long-range coulombic interactions, the particle–particle particle–mesh (pppm) solver was applied with an absolute charge value of 10^−5^. The cut-off radius for the short-range and coulombic interactions was set to 11 Å. To remove the memory of the initial configuration, the system was maintained at 3000 K for 10 ps and 100 ps with NVT and NPT ensembles, respectively. Afterward, the system was cooled to 1973 or 1823 K at a rate of 1 K/ps with the NPT ensemble. Finally, to achieve an equilibrium state, the system was relaxed at 1973 or 1823 K for 200 ps and 200 ps with NPT and NVT ensembles, respectively.

## Supplementary information

Supplementary Information
