# Supplementary material for: Estimation of TiO2-FeO-Na2O slag viscosity through molecular dynamics simulations for an energy efficient ilmenite smelting process
Source: Sci Rep. 2019 Nov 22;9:17338. doi: 10.1038/s41598-019-53961-1 (PMC6874600; doi:10.1038/s41598-019-53961-1)
Supplement: Supplementary file 1 — Supplementary Information [file 41598_2019_53961_MOESM1_ESM.pdf]

Supplementary Information

Estimation of ilmenite smelting slag viscosity through molecular dynamics simulations for an energy efficient process

AUTHOR LIST AND AFFILIATIONS

Youngjae Kim and Hyunsik Park*

Mineral Resource Research Division, Korea Institute of Geoscience and Mineral Resources (KIGAM), Daejeon 34132, Republic of Korea

*E-mail Address: Hyunsik.Park@kigam.re.kr (H. Park)

Supplementary Table S1. Measured viscosity (mPa-s) of present $FeO-TiO_2$ and $FeO-TiO_2-Na_2O$ system with varying temperature.

	Composition (mol%)			Temperature (K)												
	FeO	TiO_2	Na_2O	1823	1798	1773	1748	1723	1698	1673	1648	1623	1598	1573	1548	1523
1	60	40		7.88	7.45	8.90	10.38	-	-	-	-	-	-	-	-	-
2	50	50		11.03	11.19	-	-	-	-	-	-	-	-	-	-	-
3	40	60		16.43	16.49	-	-	-	-	-	-	-	-	-	-	-
4	38.8	58.2	3.0	15.78	14.53	13.98	14.43	14.90	15.87	16.89	17.14	-	-	-	-	-
5	37.6	56.4	6.0	13.95	14.84	15.95	17.26	17.62	17.71	17.73	18.13	18.27	18.39	458.04	-	-
6	36.4	54.6	9.0	10.24	10.97	11.23	11.27	11.77	11.98	12.28	12.60	13.13	13.90	14.56	149.87	-
